# Recent Progress in Microencapsulation of Active Peptides—Wall Material, Preparation, and Application: A Review

**DOI:** 10.3390/foods12040896

**Published:** 2023-02-20

**Authors:** Mengjie Li, Quanyou Guo, Yichen Lin, Hairong Bao, Song Miao

**Affiliations:** 1 College of Food Science & Technology, Shanghai Ocean University, Shanghai 201306, China; 2 East China Sea Fisheries Research Institute, Chinese Academy of Fishery Sciences, Shanghai 200090, China; 3 Teagasc Food Research Centre, Moorepark, P61C996 Fermoy, Ireland

**Keywords:** active peptides, microcapsules, wall material, microencapsulation technology, application of peptides microcapsule

## Abstract

Being a natural active substance with a wide variety of sources, easy access, significant curative effect, and high safety, active peptides have gradually become one of the new research directions in food, medicine, agriculture, and other fields in recent years. The technology associated with active peptides is constantly evolving. There are obvious difficulties in the preservation, delivery, and slow release of exposed peptides. Microencapsulation technology can effectively solve these difficulties and improve the utilization rate of active peptides. In this paper, the commonly used materials for embedding active peptides (natural polymer materials, modified polymer materials, and synthetic polymer materials) and embedding technologies are reviewed, with emphasis on four new technologies (microfluidics, microjets, layer-by-layer self-assembly, and yeast cells). Compared with natural materials, modified materials and synthetic polymer materials show higher embedding rates and mechanical strength. The new technology improves the preparation efficiency and embedding rate of microencapsulated peptides and makes the microencapsulated particle size tend to be controllable. In addition, the current application of peptide microcapsules in different fields was also introduced. Selecting active peptides with different functions, using appropriate materials and efficient preparation technology to achieve targeted delivery and slow release of active peptides in the application system, will become the focus of future research.

## 1. Introduction

Peptides are organic compounds formed by the condensation of two or more α-amino acids through peptide bonds, and they can be synthesized in all cells. Active peptide fragments can be used in a variety of ways to regulate the organism or to provide nutrients for the growth and development of the body [1]. The quality of such biologically active functional peptides is usually high. The molecular weights of these active peptides are usually less than 6000 Da, and their specific functions are determined by the amino acid types and sequences, including antioxidant [2], hypotensive [3], anti-bacterial [4], anti-thrombotic [5], hypoglycemic [6], immunomodulatory [7], mineral absorption promoting [8], anti-tumor [9], anti-radiation [10], etc. Active peptides can be derived from animals, including reptiles [11], cattle [12], fish [13], egg [14], drosophila [15], shrimp [16]; plants, including soybean [17], corn [18], wheat [19], chickpea [20]; and microorganisms, including yeast [21], fungi [22], mushroom [23], and spirulina [24]. Due to their high safety, wide source, and significant effects, active peptides have good application prospects in food, medicine, agriculture, and other fields, including in the priority development field by the National Development and Reform Commission in 2017. However, naked peptides are easily affected by temperature, humidity, light, and other environmental factors in the natural environment. In addition, factors such as pH and enzymes in the internal environment of the body will also make the naked peptides unable to play their best role. All of these make their practical applications limited [25].

Microencapsulation technology can improve the utilization rate of the active peptides. Microencapsulation technology is a kind of encapsulation technology based on nanocarriers, which has gradually become a popular means to protect bioactive agents in recent years. The microencapsulation of peptides refers to the selection of appropriate wall materials and the use of physical, chemical, or physicochemical methods [26] to embed the active peptides, in order to give play to the advantages of isolating the interaction between the active peptide and the external environment. A lot of research shows that microencapsulation structure has significant effects on maintaining the activity of functional peptides, burying the undesirable odor, improving the adsorption of active peptides, improving the stability, controlling the release, etc. [27,28]. In this paper, the research progress of active peptide microcapsules in recent years was reviewed, and the wall materials commonly used in peptide microcapsules were analyzed. Compared with traditional natural wall materials, the polymer wall materials prepared by modification or synthesis showed a better embedding effect, a controlled release effect, and mechanical strength. Four kinds of microcapsule preparation technologies are introduced, among which, fluid control technology is the representative of the new technology, which can accurately control the microcapsule size, realize the mass production of microcapsules, and avoid the damage caused by high temperature to the active substance. The practical application effects of microencapsulated peptides in different fields are summarized, in order to provide reference for technological innovation, industrial production, and application of microencapsulated peptides.

## 2. Wall Materials of Peptide Microcapsules

Microcapsules are a combination of wall and core materials, and wall materials with different properties will have different effects on the physicochemical properties of core materials [29], which also involves the selection of subsequent preparation methods. The wall material is generally required not to react with the core material, has the mechanical strength to protect the core material, and has certain solubility, fluidity, emulsification, stability, etc. [30]. Therefore, it is important to choose a suitable and economical wall material, according to the characteristics of the core material and the actual application. Polymer materials are the most commonly used wall materials in microencapsulation, including natural polymer materials, naturally modified polymer materials, and completely synthetic polymer materials [31]. The use of relevant materials in active peptides microcapsules is summarized in the following section.

### 2.1. Natural Polymer Wall Materials

Common natural polymer wall materials can be divided into carbohydrates, proteins, and lipids [32]. Such materials are widely used in the preparation of peptides microcapsules, due to their broad variety of natural sources, non-toxicity, non-irritation, good biocompatibility, and film-forming properties.

#### 2.1.1. Polysaccharides

Polysaccharides are green, nutritious, and healthy macromolecules, containing monomers with only three elements (carbon, hydrogen, and oxygen), which are polymerized through glycosidic bonds. Among them, sodium alginate, chitosan, pectin, and cellulose have promising applications in drug delivery.

Alginate is a natural polysaccharide extracted from brown algae or bacterial cell walls. Due to its good solubility, biocompatibility, gelation, ease of film formation, and natural non-toxicity, alginate has been widely used in the preparation of antibacterial films, dressings, microcapsule wall materials, hydrogels, and other aspects [33,34,35]. Calcium chloride solution is often added to sodium alginate, and alginate and metal particles are complexed to form calcium alginate gel systems to obtain stronger toughness and hardness. When Ca^2+^,-COO^-^ and -OH work together to provide coordination bonds for other molecules in the system, the “egg box”-shaped three-dimensional network structure has a positive effect on the encapsulated active substance [36]. By increasing the temperature, the chain structure of sodium alginate can be expanded and complexed with Ca^2+^; when the temperature is too high, the structural unit of sodium alginate is destroyed, which is not conducive to the formation of the condensation system. The α-calcitonin gene-related peptide (α-CGRP) is a neuromodulatory peptide widely distributed in the central nervous system, which has a vasodilatory function and plays an important role in the pathogenesis of migraines. Its biological half-life is about 18 min, which means the time required to reduce the α-CGRP dosage in the body or the drug concentration in the blood by half is 18 min. Kumar et al. [37] studied alginate-based α- CGRP microcapsules and tested the efficacy of alginate -α-CGRP microcapsules in a TAC pressure overload heart failure mouse model. The results showed that alginate-α-CGRP microcapsules effectively prolonged the release of the peptide, while administering mice with significantly reduced cardiopulmonary weight, left ventricular cardiomyocyte size, apoptosis, and increased oxidative stress. Oki et al. [38] developed a sulfhydryl-maleimide-modified alginate microcapsule, and the modified alginate could be encapsulated by “in situ conjugation” of sulfhydryl-containing peptides. These in situ binding methods of functional peptides have promising applications in biomedicine, chemistry, and other aspects.

Chitosan is also a kind of natural polymer material that is obtained by removing part of the acetyl group from chitin. It is biodegradable, biocompatible, non-toxic, hygroscopic, and film-forming and has various physiological functions, such as antibacterial, anticancer, lipid-lowering, etc., as the only alkaline polysaccharide among natural polysaccharides [39]. Zhao et al. [40] prepared microcapsules of desalinated duck egg white peptide calcium (DPS-Ca) with chitosan, which can prevent DPS-Ca from being digested by the stomach and make more calcium entering the intestine, promoting calcium absorption. Furthermore, since chitosan contains chemically modifiable -NH2 and -COOH, when dissolved in dilute acid, it will produce primary amino acids, which makes the molecular chain of chitosan carry a large amount of positive charge; the molecular chain of sodium alginate carries a large amount of negative charge in aqueous solution when a polyelectrolyte film is formed between the two through electrostatic interaction. Owing to the fact that this method does not require high temperature and chemical force for the preparation of films, it is an ideal encapsulation material for active substances, such as proteins and peptides, that are easily degradable and have a short half-life [41]. Salvatore et al. [42] constructed chitosan sodium alginate hollow capsule shells using the jet method and the layer-wise self-assembly method, in which fluorescently labeled peptide substrates were involved in the sortase-catalyzed trans-peptide reaction.

In addition, some polysaccharide gums from plants are also ideal materials for embedding active peptides. L-rhamnose, D-galactose, arabinose, etc., constitute these polysaccharide gums. The most widely used are Arabic gum, pectin, algae gum, and locust bean gum [43]. Different gums have different physical and chemical properties. Algae gum, for example, is a low-fat, high-dietary fiber polysaccharide material, including agar and alginate. Hambleton et al. [44] studied the interface characteristics of microcapsule products covered with carrageenan and found that the microcapsule of carrageenan wall material has the characteristics of a uniform surface, smoothness, and high strength. Joanna et al. [45] constructed the antioxidant peptides microcapsules embedded by algae gum and evaluated the lipid distribution, antioxidant blood status, and mRNA expression of some genes related to antioxidant status in rats in vitro experiments and confirmed that the serum level of total antioxidant status was significantly increased in animals that consumed microcapsules without causing oxidative stress. Raúl E et al. [46] found that acacia bean gum and algae gum as composite wall materials had better encapsulation rates and resistance to digestive enzymes for ACE-I inhibitory peptides, compared to both of them used independently.

Dextrins are oligosaccharides formed by the physical, chemical, or enzymatic decomposition of starch, of which, cyclodextrins are small glucose molecules with cavities in rings formed by straight-chain starch under the action of enzymes produced by Bacillus, with the advantage of non-toxic and easily absorbed by humans. The structural characteristics of cyclodextrins allow them to form a complex structure by combining specific compounds of compatible size in an aqueous solution to achieve the functions of stabilization, sustained release, oxidation resistance, and odor masking of the object [47]. β-cyclodextrin is an ideal wall material for hydrophobic substances, due to the hydrophilic properties of the outer wall and hydrophobic properties of the inner cavity [48]. The nano-sponge is a colloidal structure composed of solid nanoparticles with holes and a reticular structure, which is used to encapsulate various substances. The β-cyclodextrin-based nano-sponge has potential application value in the targeted drug delivery of the liver, spleen, and lung and is an ideal carrier for protein and polypeptide drug delivery [49]. Neuropeptide Y (NPY) is a hormone with a stabilizing effect on the human body. Desai et al. [50] aimed to compound NPY with hydroxypropyl β-cyclodextrin (HPβ-CD) to finally achieve NPY microcapsules with an encapsulation rate of 84.68 ± 5.47% and a 24 h release of 85.16 ± 6.13% in vitro.

#### 2.1.2. Protein

Protein is an appropriate wall material for embedding active substances, due to its good biocompatibility and biodegradability, and is regarded as “Generally recognized as safe” by the FDA [51]. Soybean protein, gelatin, whey protein, and zein can be used as the embedding materials of active peptides. Different proteins have different abilities for drug loading and controlled release, due to their different properties, structures, and functions. However, the spontaneous aggregation behavior of proteins and the protease in the body can both damage the structure of microcapsules. The combination of protein and other materials can optimize the embedding effect [52].

Gelatin is a macromolecular colloid produced from the decomposition of animal collagen. Its amphiphilic makes it effectively act as a binding agent between composite walls and between walls and core materials. Compared with plant proteins, animal proteins have better film-forming properties [53], considered a high-quality natural encapsulation carrier. Nevertheless, the application of gelatin is often limited by its low mechanical properties and poor bio-adhesiveness, and it is usually used in the form of hard gelatin capsule shells. These kinds of defects can be optimized by compounding with other materials to achieve their wall properties [54,55]. Favaro et al. [56] prepared a gelatin compounded with soybean isolate as the wall material, and the peptides microcapsules were proved to be more stable and to have lower bitterness than the raw peptides. According to Niu et al. [57], the composite wall material of gelatin and sodium alginate was used to embed methionine, which realized the controlled release of methionine and simultaneously stimulated the synchronous absorption of other amino acids to synthesize proteins.

As a common protein of plant origin, soy protein has an amino acid composition similar to that of milk protein and has a nutritional value equivalent to that of animal protein, which also has isoflavones linked to lower cholesterol levels [58]. Through industrial processing, soy protein flour (SPF), soy protein concentrate (SPC), soy protein isolate (SPI), and tissue soybean protein (TSP) exhibit remarkable emulsification and film-forming properties, and their unfolding structure in the emulsion wrap around the oil phase to stabilize the water–oil interface. It is worth mentioning that SPI has high protein concentration, emulsification, and cohesion, and the macromolecular complex formed by electrostatic self-assembly with acid-resistant SPSS is a good wall material for embedding hydrophobic active substances. It has been shown that the pre-heating treatment of SPI results in stronger emulsification and the interfacial elasticity of it, as well as tighter complexation with polysaccharides; moreover, heating exposes more internal hydrophobic groups by unfolding the structure of SPI, which is beneficial to the embedding of hydrophobic peptides [59]. Wei et al. [60] utilized the self-assembly ability of protein hydrolysate and polyphenol curcumin to mask the bitter hydrophobic residue of protein hydrolysate through hydrophobic combinations. By embedding with wall material SPI and SPC, the soybean peptide–curcumin nanoparticles microcapsules were prepared with an embedding rate of 50.92%, bitterness reduction of 2.68 times, and moisture absorption reduction of 1.61 times.

Whey protein is a high-quality protein that is easily absorbed, low in sugar, and has the highest nutritional value of all proteins. Industrial whey proteins are mainly derived from by-products of cheese production and are dominated by whey protein concentrate (WPC) and whey protein isolate (WPI). Whey protein has high nutritional value, and as the wall material of microcapsules, it also has pH-sensitive characteristics specific to the controlled release of the cores [61]. Considering whey as a natural bacterial medium, Farizano et al. [62] investigated the use of WPC and chitosan derivatives as composite wall materials to encapsulate Gram-positive bacteria with bacteriocin-erythromycin CRL35, which not only maintained bacteriocin activity, but also provided a scheme for the green treatment of whey.

#### 2.1.3. Lipids

**Figure 1:**
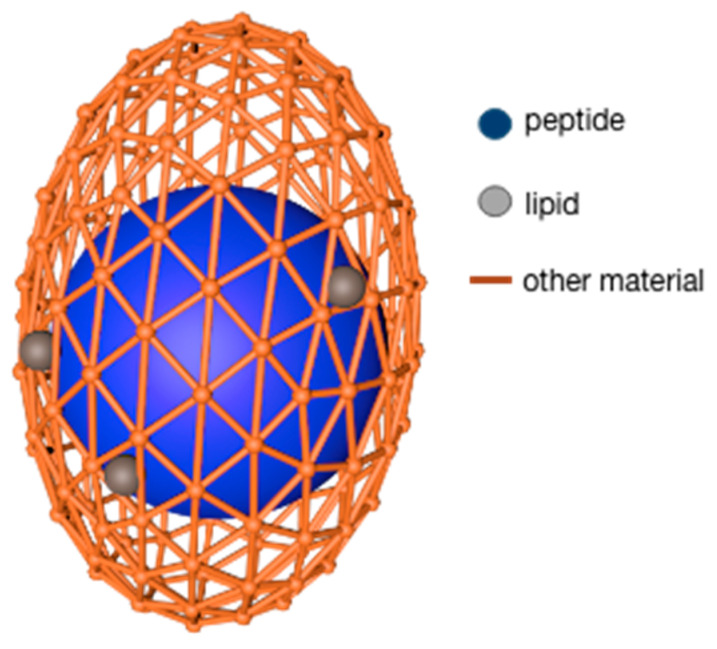
Microcapsule wall materials of alginate filled by lipid.

The lipids used as microcapsule wall materials are generally fatty acids and lecithin. The advantages of this type of wall material are that it can encapsulate both hydrophilic and lipophilic core materials and it has good biocompatibility, and targeting, among which, stearic acid also shows good oxidation resistance. Despite so many benefits, lipids still have some disadvantages: the preparation efficiency is not high, and the preparation method is limited by the properties of lipids [63]. N. Blanco-Pascual et al. [64] used a mixture of stearic acid and Brazilian carnauba wax (3:1) as a wall material and peptide solution as a core material to produce microcapsules with a shell material/peptide dry base ratio of 13.3:1. This product was realized by the microencapsulation printing technique. The microcapsules were found to have good stability (<30% peptides release within 3 h) under experimental conditions of pH5 and pH7. Further research revealed that the modifying properties of lipids and the chemical interaction with other biopolymers both allow lipids to be worked as wall fillers to promote the formation of dense walls; at the same time, the lipid–peptide complex makes the core material more stable, similar to the model in Figure 1 [63].

### 2.2. Modified Polymer Wall Materials

Through the modification of natural materials, the mechanical strength and stability of natural materials are enhanced, and the limitation of the application of natural materials is made up, which makes natural materials a good tool to protect the inner substance. Researchers usually use modified protein or modified cellulose as the shell of the microencapsulated peptides.

#### 2.2.1. Modified Cellulose

Cellulose is the most popular food packaging material now—it is non-toxic, biodegradable, and has good barrier properties, making it a substitute for traditional petroleum-based plastics. The hydrophilicity of cellulose is the biggest limitation as a packaging material, which can be solved by modifying it. The researchers reported, in detail, the cellulose modification technology and the application prospect of chemically modified cellulose in food packaging [65]. The modified cellulose used for embedding peptides is introduced below.

Hydroxyethyl cellulose (HEC), hydroxypropyl methyl cellulose (HPMC), carboxymethyl cellulose (CMC), and nanocellulose are all common materials for embedding peptides. As early as 1999, Ohyama et al. [66] tried to embed islets with agarose, polystyrene sulfonic acid, and polybrene carboxymethyl cellulose to realize xenotransplantation of islets. Microencapsulation proved to be successful in prolonging islet survival. Up until now, great progress has been made in embedding delivery materials for islets of Langerhans [67]. Cesar et al. [68] prepared Ctx(Ile21)-Ha antimicrobial peptide microcapsules encapsulated by hydroxypropylmethyl cellulose phthalate (HPMCP) as an alternative to traditional antibiotics and as a production enhancer for chick rearing.

#### 2.2.2. Modified Protein

The modification of amino acid components, charged properties, and spatial structure of proteins through physical, chemical, enzymatic, or genetic engineering is called protein modification, leading to a broad foundation for the application of proteins in many fields [69,70,71]. The modified proteins are less sensitive to the environment and obtain better stability, emulsification, and solubility.

Wang et al. [72] studied the chemical, enzymatic, and physical modifications of rapeseed isolate protein (RPI) to improve its mechanical properties as a wall material for spray-dried rapeseed peptide (RP) microcapsules. The experimental results showed that both acylation of the wall material RPI (acylation degree (DA) of 47%) and high pressure (HP) of 400 MPa resulted in greater encapsulation of microcapsules, with 99% and 94%, respectively.

### 2.3. Synthetic Polymer Wall Materials

The synthetic polymer materials used for peptide microcapsules wall materials mainly include degradable polymers, polyesters, polydextrose, and non-degradable polymers (polyacrylamides). Such materials have stronger manipulability and specificity and have become potential materials in the medical, biological, and food fields in recent years. Polymer-embedded microcapsules tend to have a more homogeneous particle size and higher mechanical strength for a wide range of applications.

#### 2.3.1. Polylactic Acid

**Figure 2:**
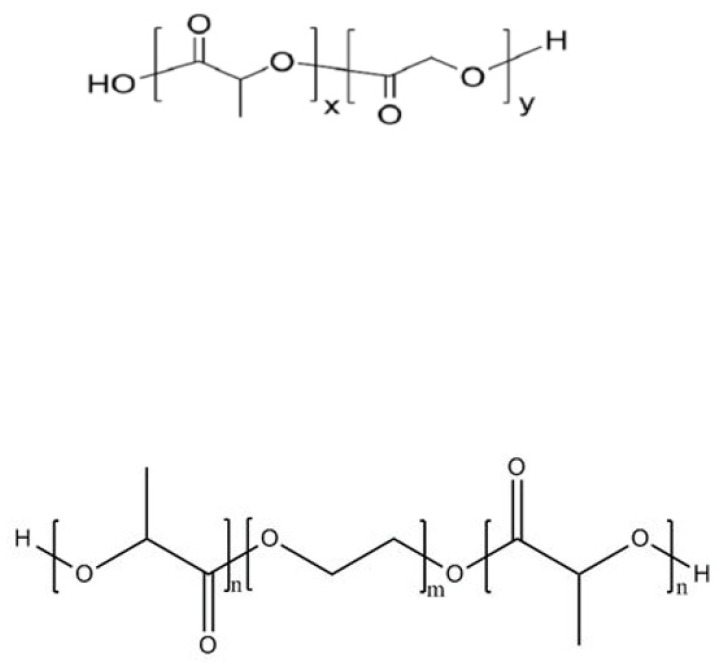
Molecular structure formulas of PLGA (up) and PLA-PEG-PLA (down).

Polylactic acid (PLA), a polymer formed by dehydration of lactic acid (LA), is the most widely used polyester carrier for protein and peptide drugs, due to its adjustable molecular weight and good biocompatibility. It is often used in copolymers with other materials (e.g., PLGA and PLA-PEG-PLA) (Figure 2). PLGA carriers act as “delayed” excipients: when PLGA degrades, it produces acidic degradation products that cause a drop in the internal pH of the polyacrylate matrix. The decrease in pH value weakens the peptides–polymer interaction and enables the release of the peptides [73,74]. Justin et al. [75] investigated the thermodynamics of cationic octapeptide octreotide binding to PLGA using nanoisothermal titration calorimetry (NanoITC), revealing that the actual driving force for octreotide-PLGA interaction is ion pairing. Cationic peptides readily bind PLA-PLGAs to carboxylic acid (-COOH) end groups, and such binding is key to leading to PLGA-based peptide acylation, which poses a significant challenge for both drug microencapsulation and delivery systems. Lim et al. [76] found that the encapsulation of exenatide acetate in PLGA microspheres using the *w*/*o*/*w* double-emulsion solvent evaporation method and the determination of peptide release profiles and acylation products of PLGA polymer degradation in PLGA microspheres showed that this method significantly improved the stability of exenatide acetate. Meanwhile, the acylation of the PLGA polymer was eliminated during the degradation process in vitro, and the immunogenicity was reduced in vivo. PEG is a polyether polymer with a flexible hydrophilic surface, which has the function of extending the circulation time and retaining the half-life of nanoparticles as a microcapsular wall material. Zhang et al. [77] used thymopentin as a model drug and the PLA-PEG (polyethylene glycol)-PLA triblock copolymer as a carrier to prepare microcapsules for cancer therapy, and this dense shell structure significantly delayed the abrupt release of the core material, which has good prospects for application as an anticancer drug.

#### 2.3.2. Polydextrose

Polydextrose (PD) is a water-soluble dietary fiber produced by the vacuum condensation of glucose and small amounts of sorbitol, citric acid, or phosphoric acid in the molten state, and it is not toxic to humans [78]. Oligoglucose with a molecular weight of 162–5000 has a regulatory effect on human intestinal flora and is most widely used in practice. Marilia et al. [79] used PD and maltodextrin (MD) polymers as wall materials and ferrous sulfate with organic peptides as core materials and improved the absorption of iron in humans by the spray drying method of encapsulated microcapsules.

#### 2.3.3. Polypropylene

Polypropylene is a common package material, and polymeric materials prepared by polymerization with its derivatives or reaction with other substances are widely used in microcapsules, hydrogels, flocculants, etc.

The hepatitis B surface antigen (HBsAg) is a mixture of peptides, and oral delivery is one of the most effective ways to deliver it. However, factors such as intestinal pH and bacterial flora pose challenges to drug delivery. In this study, HBsAg was manually filled into the commercial hard gelatin microcapsule shell, and then Eudragit S100 and Eudragit L100 (4:1 *W*/*W*) were used as the secondary coating for embedding the microcapsules [54]. The experimental results confirmed that HBsAg microcapsules constructed by this method achieved targeted delivery in the colon and produced considerable antibody volume. Gunay et al. [80] studied a peptides microcapsule with the poly(N-(2-hydroxypropyl) methacrylamide) (PHPMA) copolymer as the aromatophile and polyurethane as the microcapsule shell, and the experimental results showed that the incorporation of peptides increased the deposition of PHPMA copolymer by 3.5–5.0 times and enhanced the deposition of the fragrance system in hair.

### 2.4. Potential Material

In addition to the materials mentioned above, several substances with potential as active peptide wall materials are provided below.

**Figure 3:**
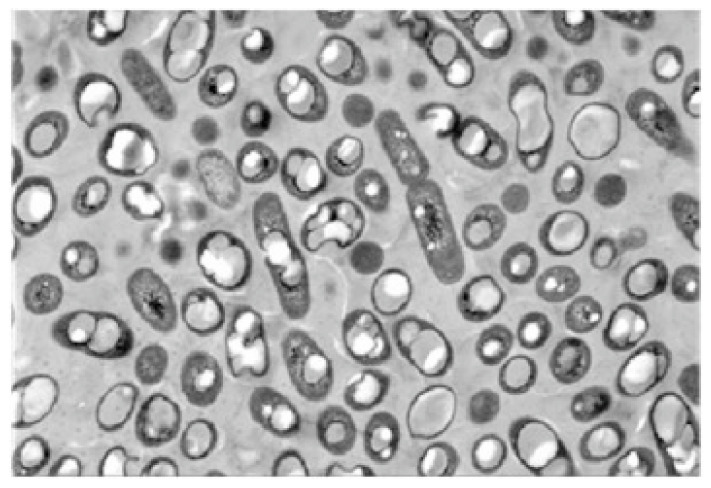
Microscopic image of PHA granules [82].

Polyhydroxyalkanoates (PHA) and their derivatives (PHB, PHO, etc.) are a kind of natural polyester materials synthesized by microbial fermentation from carbon sources, which can be degraded both in and out of cells quickly [81]. Compared with other natural materials, polyhydroxyalkanoates exhibit better high-temperature stability, lower surface porosity, and enhanced toughness and elasticity, making them popular in the medical field as raw material of nanoparticles (Figure 3) [82]. In the study, Cao et al. [83] encapsulated trifluralin with PHB polymer as the carrier, and the microcapsules formed showed better photostability and herbicidal activity. The release rule could be adjusted by changing the preparation parameters, such as the shear rate and emulsifier concentration.

Inorganic materials usually have good chemical and thermal stability, so they are often used in the field of biodegradation and environmental protection [84]. Among them, bimetallic hydroxide, calcium carbonate, and phosphate can be used as microcapsule wall materials. Hollow microcapsules show good performance in drug delivery and personalized medicine. However, microcapsules made of polyelectrolytes are soft and unstable in harsh environments. Jin et al. [85] reported an improved Ca^2+^ cross-linked hard-shell microcapsule to improve the instability of microcapsules made of polyelectrolytes in harsh environments. The experimental results confirm that the Ca^2+^ embedded hard-shell microcapsules have good mechanical strength and a two-stage sustained release effect.

Chitosan derivatives are also potential materials for embedding active peptides. Modified chitosan has better biocompatibility and a controlled release effect. Chatterjee et al. [86] prepared fish oil microcapsules using n-lauryl chitosan modified by n-acylation as the wall material. The resulting microcapsules had high loading capacity and a good slow-release performance, which was achieved through core material diffusion, rather than wall material disintegration. Lauryl acylation improves the hydrophobicity of chitosan and reduces the nucleophilicity of free amine groups in chitosan. This property can be used for embedding lipid soluble polypeptides. The information of different classification of materials has been shown in Table 1.

## 3. Preparation Technology of Peptide Microcapsules

The preparation technology of microcapsules has been innovated with the expansion of microcapsule application fields. The traditional preparation methods include physical methods, such as spray drying methods and electrostatic binding methods; chemical methods, such as sharp pore methods and interfacial polymerization methods; physicochemical methods, such as aqueous phase separation methods and oil phase separation method. The above traditional methods have been reported adequately [93,94,95]. More details have are shown in Table 2. Four novel or efficient techniques for the preparation of peptide microcapsules will be introduced in this section.

### 3.1. Micro-Controlled Flow (Microfluidic) Method

**Figure 4:**
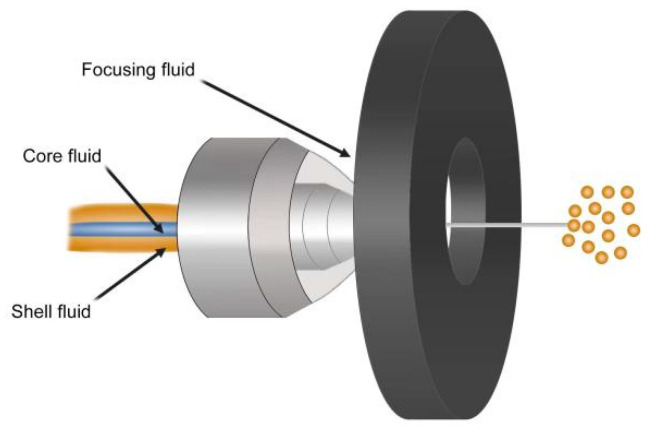
Preparation of nano microcapsules by microfluidic.

Microfluidic methods are considered to have great potential in the field of biopharmaceuticals. When microcapsules are prepared by the Microfluidic (Capillary microfluidic device from World Precision Instruments, Inc. manufacturer: Sutter Instrument Co., Novato, CA, USA), two-phase liquids with different flow rates are produced by the injection pump and enter their respective micro-pipelines. Then, the liquids with different behaviors meet in the micro pipelines, and they will form uniform droplets, due to the action of interfacial tension and shear force, to realize the homogenization and emulsification of the wall and core materials [104], while forming nanoemulsions (Figure 4). The advantages of microcapsules prepared by this method have a regular structure and good embedding effect, and the height of microcapsule diameter can be selected by adjusting parameters, such as flow rate, microtubule size (nanoscale), and other parameters. However, the preparation of microcapsules by micro-controlled flow technique is costly and cannot be achieved in mass production, so this technique is suitable for projects requiring high precision.

Shimanovich et al. [105] prepared stimuli-responsive microcapsules of amyloid peptides with pH change-triggered release characteristics based on this method, which is biocompatible and biodegradable and can be used as a controlled release model for a variety of biomolecules. Han et al. [106] found a significantly longer half-life of microencapsulated peptides and higher pulmonary distribution of the drug, compared to peptides alone, by micro-controlled flow-encapsulated innate immune-targeted hexapeptide nanoparticles. These novel nanoparticles with lipid shells have considerable potential to increase the circulating half-life and improve the biodistribution of therapeutic peptides to enhance their clinical utility, including those aimed at treating lung-related diseases.

### 3.2. Microjet Method

The microjet (high-pressure micro-jet homogenizer from China, ATS Industrial System Co., Ltd. manufacturer: Genizer, USA.), simply speaking, is to suck the premixed core-wall composite solution into the microfluidizer through a slit, and the reciprocating cavity in the microjet gives the composite solution ultra-high pressure, so that the composite solution reaches the sound speed. The composite solution loses pressure instantly at the exit of the slit, resulting in the cavity effect, impact effect, and shear effect, in order to realize the transition from composite solution to nanoemulsion, and then the nanoemulsion is dried to prepare microcapsules. The microcapsules produced by this method have small and regular particle sizes (nanoscale), homogeneous structures, regular morphology, and a good embedding effect. However, due to the limitations of the apparatus, the preparation of microcapsules by this method requires that the solid content of the composite should not be too high, and the process of microjet flow will generate heat, which may lead to some influence on the active material. As a good emulsification method for core/wall materials, the microjet has developed rapidly in the field of microencapsulation in recent years, but there are few reports on the microencapsulation of protein and peptide drugs [107,108,109]. Alfonso et al. [110] systematically described how to encapsulate small molecules, such as proteins, in microcapsules of controllable size by using microjet technology.

### 3.3. Layer-by-Layer Self-Assembly (LBL) Method

The LBL method is a technique to prepare microcapsules based on charged templates by alternating layers of positive and negative ions on the templates to form thin films or microcapsules and subsequently removing the templates. The advantage of this method is that the damage to the packaged object is small, but it cannot be mass-produced. Laura et al. [111] constructed antimicrobial peptide microcapsules with diameters of about 2 μm, based on chitosan/alginate encapsulation, as a novel antimicrobial coating for medical cotton gauze.

### 3.4. Yeast Cell Wall Method

Researchers used wine yeast as the raw material of the wall and then dissolved the cell contents by acidic and enzymatic digestion. The obtained yeast cavity was in constant contact with the core material to realize the embedding of the core material. Yeast cell wall microcapsules are stable in the acidic and enzymatic environment of gastric juice, which can effectively protect drugs from passing through the gastric environment [112]. In addition, the yeast cell wall itself contains dextran and mannan oligosaccharides, which can improve the resistance of the human body to diseases and regulate the stability of the microbial environment. Huizar et al. [113] used dextran particulate yeast cell wall-embedded lipoprotein peptides for injection, and the drug entered the organism to induce an effective Th17 cell response, without causing a strong tissue reaction at the injection site.

## 4. Application of Peptides Microcapsules

### 4.1. Application of Peptides Microcapsules in Biopharmaceuticals

Microencapsulation technology aims at drug encapsulation and with the realization of microcapsules with nanoscale particle size, and its application has been extended to target control of anticancer drugs, sustained release, oral vaccines, and the immobilization of cells and enzymes.

Structured nanoengineered antimicrobial peptide polymers (SNAPPs) are an emerging class of antimicrobial agents targeting multi-drug resistant bacteria. Song et al. [92] constructed SNAPPs microcapsules by two different methods and demonstrated their potential application in the pulmonary delivery of antimicrobial drugs against respiratory bacterial infections (e.g., pneumonia and tuberculosis).

The subcutaneous transplantation of islet microcapsules has been extensively studied as a treatment for type 1 diabetes. However, due to the low density of subcutaneous vessels and the strong inflammatory response, the successful normalization of blood glucose levels has rarely been reported. To address this problem, Mochizukiet et al. [114] developed a mosaic-like aggregate composed of mesenchymal stem cells (MSCs) and recombinant peptides that sustained the release of angiogenic factors and anti-inflammatory cytokines. The aggregates were co-microencapsulated with rat islets and subcutaneously transplanted into mice with an immunoreactive diabetes mellitus model. By observing the glycemic changes 28 days after transplantation, the normalization rate of the novel microcapsules was found to be significantly higher than that of MSC cell-free microcapsules and MSC cell microcapsules transplanted outside the microcapsules, demonstrating the superior effect of the novel microcapsules over unreformed microcapsules.

The oral administration of live probiotics as antigen-delivery vehicles is a promising approach to vaccine development. However, the low survival rate of probiotics in the gastrointestinal tract limits this approach. Jiang et al. [115] used recombinant Lactobacillus plantarum 25 (LP25) expressing an M-cell homing peptide fused with BmpB protein as a model probiotic, and the microencapsulated probiotic survived >65% in simulated gastric fluid (pH 2.0) and >75% in simulated small intestinal fluid (pH 7.2) and was completely released within 12 h. Four weeks after the first immunization, the drug induced a stronger production of BmpB-specific IgG and IgA in mice and proved to be a promising delivery system.

### 4.2. Application of Peptide Microcapsules in Food

Peptide microcapsules with specific functionalities can function as health ingredients on their own or in combination with other edible components.

Zhao et al. [40] encapsulated the egg white peptide calcium (DPS CA) chelate into microcapsules, which promoted the absorption of calcium in calcium-deficient mice and improved the intestinal flora of mice. This model has good prospects in the delivery of functional food.

Nisin, as a natural antibacterial peptide, is widely used as a preservative in the food field. To solve the problem of reduced activity of nisin in the food system, Diamante et al. [27] encapsulated nisin in the form of microcapsules. The results showed that, under the experimental conditions of 4 °C and pH 6.0, the microcapsule structure could effectively protect nisin from the influence of protease within 24–168 h. In addition to the oral delivery system, peptide microcapsules with antibacterial activity can also be used for the preservation of fruits and vegetables, meat, seafood, etc. Through the slow-release characteristics of microcapsules, the controlled release of antibacterial peptides during food storage can be realized. Wang et al. [116] studied the effect of the brevilaterin antibacterial peptide after microencapsulation, showing that brevilaterin antibacterial peptide microcapsule has an obvious antibacterial effect on golden grape ball plate, and the effect of 3000 au/g brevilaterin antibacterial peptide microcapsule on bread preservation is better than 1 g/kg calcium propionate, indicating that the antibacterial peptide microcapsule has a considerable effect on food preservation.

### 4.3. Application of Peptide Microcapsules in Other Fields

With the variety of functional peptides and the continuous improvement of embedding technology, researchers are also committed to developing the application of functional peptide microcapsules in various fields.

In the beauty industry, natural active peptides and biomimetic peptides have been widely used as anti-wrinkle agents to delay skin aging. Hu et al. [117] have prepared antibacterial peptide nal-p-113 nanoparticles for root caries repair. The antibacterial test shows that the prepared nanoparticles have obvious inhibitory effects on *Clostridium nucleatum*, *Streptococcus gordonis*, and *Porphyromonas gingivalis*.

As for daily chemical products, the deposition of fragrance delivery systems onto hair from a shampoo formulation is always a challenging task, and Günay et al. [80] took phage identification peptides that can bind to hair as core materials and PHPMA as a perfume carrier. After microencapsulation, the deposition of PHPMA copolymer increased by 3.5–5.0 times, increasing the deposition and release of aromatic substances on hair.

In addition, antibacterial peptide microcapsules can also be used as feed additives against pathogens. Cesar et al. [118] used antibacterial peptide microcapsules to treat laying hens infected with streptococcus enteritidis. The results showed that the amount of Streptococcus enteritidis in the liver, spleen, and cecum of laying hens was reduced by feeding antimicrobial peptide microcapsules.

Major information is shown in Table 3.

## 5. Summary

Peptide substances with biological activity are the key development objects in the fields of medicine, food, and building materials in recent years. According to different active peptide substances, the selection of suitable wall materials and preparation methods is the key to innovative microencapsulation technology. The optimization of peptide microencapsulation is mainly considered from the following three aspects: Firstly, the effect of the composite wall material is better than the single wall material. The composite wall material is more effective than the single wall material, and the functional characteristics of multiple materials as microencapsulation wall material can be realized simultaneously through the composite of wall materials to achieve a better embedding effect. Secondly, compared with the traditional spray drying method, the use of new technologies, such as flow control and the LBL method to prepare capsules, is more conducive to the protection of biological activity and accurate control of capsule size. Thirdly, suitable active peptide and microcapsule peptide preparation methods were selected for different fields and different target objects to expand the application field of active peptide. The modification of wall materials, the study of slow release and release kinetic model of microcapsules, and the mass production of high precision microcapsules are also the key issues that need to be addressed nowadays, and the creative preparation of functional peptide microcapsules for a given endpoint should become a key issue for the industry to focus on. In the following study, the modification technology of natural wall wood was deeply explored to realize the coupling of modified materials with active peptide molecules at specific sites, so as to improve the utilization rate and embedding rate of natural wall wood and the stress potential of active peptides in food and other fields. Fluidic technology reflects the significant advantages of high preparation efficiency, good embedding rate, and uniform particle size in the preparation of microcapsules. Combined with medical polymer materials, it can be widely used in the preparation of insulin drug peptide microcapsules to achieve the purpose of precise delivery and slow release of drug microcapsules in vivo.

## Figures and Tables

**Table 1:** Classification of wall materials of peptides microcapsules.

Classification	Source	Common Types	Advantages	Disadvantages	References
Natural polymer wall materials	Extracted from natural substances	Carbohydrates. protein, lipids	Biocompatibility, environmentally friendly and wide range of sources at low cost	Low mechanical strength, low loading capacity	[33,34,35,36,39], [49,50,51,52], [87]
Modified polymer wall materials	Modification of natural material	Modified protein, modified cellulose	Good stability and Does not age easily	Difficult to prepare, low preparation efficiency	[59], [61,62,63], [65]
Synthetic polymer wall materials	Artificial synthesis	Biodegradable, non-biodegradable	High drug loading capacity, high mechanical strength	Expensive	[63,65] [69] [88,89,90]
Metallic material	-	Au, Fe3+	Good stability	Low drug loading capacity	[91,92]

**Table 2:** The traditional preparation methods for peptides microcapsule.

Method	Equipment	Advantages	Disadvantages	References
Ionotropic gelation	-	Simple operation	Time-consuming	[40,96]
Spray drying	Spray dryer	Easy operation, continuous production	Particle size heterogeneity	[97,98]
Extrusion–spheronisation	Constant flow pump	Low temperature	Low production rate	[99,100]
Molecular embedding	Ultrasonic	Easy operation	Core material limited	[49]
Solvent evaporation	Vacuum freeze dryer	Low temperature	Residual solvents	[101,102]
Free radical polymerization	-	Simple operation, low cost	Low production rate	[103]

**Table 3:** Application of peptides microcapsules in different fields.

Applications	Peptides	Objective	Function	Reference
Biopharmaceuticals	Antimicrobial peptide	Antipneumonia drug	Preventing peptide degradation, reducing clearance, enhancing intracellular delivery	[92]
	Recombinant peptides and rat islets	Subcutaneous Islet drug	Improving blood sugar normalization	[114]
	M-cell homing peptide	Oral vaccine	Improving the vitality and survival rate of peptides	[115]
Food	Duck egg white peptides-calcium	Calcium supplement	promoting calcium absorption, avoiding stomach digestion	[40]
	Nisin	Nisin storage	Improving survival rate of nisin	[27]
	Antibacterial peptide	Bread preservative	Increasing preservation time of bread	[116]
Others	Antibacterial peptide	Anti-cavity drug	Increasing bacteriostasis rate	[117]
	Phage identification peptides	Fragrance retaining agent	Increasing the deposition and release of aromatic substances on the hair	[80]
	Ctx(Ile21)-Ha antimicrobial peptide	Feed additive	Reducing systemic infection of Streptococcus enteritidis in chickens	[118]

## Data Availability

Data are contained within the article.

## References

[1] Yang F.J., Chen X., Huang M.C., Yang Q., Cai X.X., Chen X., Wang S.Y. (2021). Molecular characteristics and structure–activity relationships of food-derived bioactive peptides. J. Integr. Agric..

[2] Jose M.L., Paulo E.S., Munekata B.G., Francisco J.B., Leticia M., Cristina P.S., Fidel T. (2018). Bioactive peptides as natural antioxidants in food products—A review. Trends Food Sci. Technol..

[3] Shukla P., Chopda K., Sakure A., Hati S. (2022). Current Trends and Applications of Food Derived Antihypertensive Peptides for the Management of Cardiovascular Disease. Protein Pept. Lett..

[4] Moscoso M.G., Zavaleta A.I., Mujica Á., Arnao I., Moscoso N.C., Santos M., Sánchez J. (2021). Antimicrobial peptides purified from hydrolysates of kanihua (Chenopodium pallidicaule Aellen) seed protein fractions. Food Chem..

[5] Ding B., Xu Z.H., Qian C.D., Jiang F.S., Ding X.H., Ruan Y.P., Ding Z.S., Fan Y.S. (2015). Antiplatelet Aggregation and Antithrombosis Efficiency of Peptides in the Snake Venom of Deinagkistrodon acutus: Isolation, Identification, and Evaluation. Evid.-Based Complement. Altern. Med..

[6] Huang T.H., Liu P.Y., Lin Y.L., Tai J.S. (2021). Hypoglycemic peptide-enriched hydrolysates of Corbicula fluminea and Chlorella sorokiniana possess synergistic hypoglycemic activity through inhibiting pseudo-glucosidase and dipeptidyl peptidase-4 activity. J. Sci. Food Agric..

[7] Chen M.W., Zhang F., Su Y.J., Chang C.H., Li J.H., Gu L.P., Yang Y.J. (2020). Immunomodulatory effects of egg white peptides on immunosuppressed mice and sequence identification of immunomodulatory peptides. Food Biosci..

[8] Zhao L., Huang S.L., Cai X.X., Hong J., Wang S.Y. (2014). A specific peptide with calcium chelating capacity isolated from whey protein hydrolysate. J. Funct. Foods.

[9] Luan X., Wu Y., Shen Y.W., Zhang H., Zhou Y.D., Chen H.Z., Nagle D.G., Zhang W.D. (2021). Cytotoxic and antitumor peptides as novel chemotherapeutics. Nat. Prod. Rep..

[10] Wu Y.H., Farrag H.N., Kato T., Li H., Ikeno S. (2021). Design and Synthesis of Novel Peptides to Protect Ferulic Acid against Ultraviolet Radiation Based on Domain Site IIA of Bovine Serum Albumin. Biomolecules.

[11] Zhong H., Shi J.Y., Zhang J.H., Wang Q.Q., Zhang Y.P., Yu P., Guan R., Feng F.Q. (2022). Soft-Shelled Turtle Peptide Supplementation Modifies Energy Metabolism and Oxidative Stress, Enhances Exercise Endurance, and Decreases Physical Fatigue in Mice. Foods.

[12] Zhang H.R., Zhao L.Y., Shen Q.S., Qi L.W., Jiang S., Guo Y.J., Zhang C.H., Riche A. (2021). Preparation of cattle bone collagen peptides-calcium chelate and its structural characterization and stability. LWT-Food Sci. Technol..

[13] Mofieed A., Amit K.V., Rajan P. (2020). Collagen extraction and recent biological activities of collagen peptides derived from sea-food waste: A review. Sustain. Chem. Pharm..

[14] Miguel M., Vassallo D.V., Wiggers G.A. (2020). Bioactive peptides and hydrolysates from egg proteins as a new tool for protection against cardiovascular problems. Curr. Pharm. Des..

[15] Cynthia L., Emilie M., Joachim V.L., Lina V., Jozef V.B. (2019). Peptides in insect oogenesis. Curr. Opin. Insect Sci..

[16] Xu H.J., Chen Y.L., Li J.W., Luo J.Y., Wang Y.M., Ma W.M. (2022). A novel unique terminal ampullae-expressed insulin-like peptide in male white shrimp. Penaeus Vannamei. Aquac. Rep..

[17] Zhang C., Zhang Y.X., Liu G.R., Li W.H., Xia S.Q., Li H., Liu X.Q. (2021). Effects of soybean protein isolates and peptides on the growth and metabolism of Lactobacillus rhamnosus. J. Funct. Foods.

[18] Wang L.Y., Lei L., Wan K., Fu Y., Hu H.W. (2021). Physicochemical Properties and Biological Activity of Active Films Based on Corn Peptide Incorporated Carboxymethyl Chitosan. Coatings.

[19] Sun S.L., Zhang G.W., Mu H.Y., Zhang H., Chen Y. (2019). The mixture of corn and wheat peptide prevent diabetes in NOD mice. J. Funct. Foods.

[20] Gupta N., Bhagyawant S.S. (2021). Bioactive peptide of *Cicer arietinum* L. induces apoptosis in human endometrial cancer via DNA fragmentation and cell cycle arrest. 3 Biotech.

[21] Guo H.K., Guo S.Y., Liu H.M. (2020). Antioxidant activity and inhibition of ultraviolet radiation-induced skin damage of Selenium-rich peptide fraction from selenium-rich yeast protein hydrolysate. Bioorganic Chem..

[22] Kenichiro N., Nobuhiro K., Noriko S., Chisato Y., Hiroshi T. (2012). Synthesis and antimycobacterial activity of calpinactam derivatives. Bioorganic Med. Chem. Lett..

[23] Zhou J.J., Chen M.F., Wu S.J., Liao X.Y., Wang J., Wu Q.P., Zhuang M.Z., Ding Y. (2020). A review on mushroom-derived bioactive peptides: Preparation and biological activities. Food Res. Int..

[24] Chen Y.H., Wang F., Zhou J.W., Niu T.T., Xuan R.R., Chen H.M., Wu W. (2020). In Vivo Antifatigue Activity of Spirulina Peptides Achieved by Their Antioxidant Activity and by Acting on Fat Metabolism Pathway in Mice. Nat. Prod. Commun..

[25] Mendis E., Rajapakse N., Kim S.K. (2005). Antioxidant properties of a radical-scavenging peptide purified from enzymatically prepared fish skin gelatin hydrolysate. J. Agric. Food Chem..

[26] Gulay O., Paola F., Iolanda D.M., Jianbo X., Esra C. (2019). A review of microencapsulation methods for food antioxidants: Principles, advantages, drawbacks and applications. Food Chem..

[27] Diamante M., Annachiara D.P., Antonietta L.S., Teresa C., Francesco E., Gianluigi M. (2016). Microencapsulation of nisin in alginate beads by vibrating technology: Preliminary investigation. LWT-Food Sci. Technol..

[28] Wang Y.F., Qi W., Huang R.L., Su R.X., He Z.M. (2016). Counterion-Directed Assembly: Counterion-Directed, Structurally Tunable Assembly of Hydrogels, Membranes, and Sacs at Aqueous Liquid-Liquid Interfaces (Adv. Mater. Interfaces 5/2016). Adv. Mater. Interfaces.

[29] Santana A.A., Cano H.D.M., Oliveira R.A., Telis V.R.N. (2016). Influence of different combinations of wall materials on the microencapsulation of jussara pulp (*Euterpe edulis*) by spray drying. Food Chem..

[30] Javier D.H.L., Luis A.B.P., Alvarez R.J., Hugo S.G. (2017). Microencapsulation using starch as wall material: A review. Food Rev. Int..

[31] Yang M.Y., Liang Z., Wang L., Qi M., Luo Z.S., Li L. (2020). Microencapsulation Delivery System in Food Industry-Challenge and the Way Forward. Adv. Polym. Technol..

[32] Jéssica S.R., Cristiane M.V. (2021). Microencapsulation of natural dyes with biopolymers for application in food: A review. Food Hydrocoll..

[33] Ramprakash B., Incharoensakdi A. (2022). Alginate encapsulated nanobio-hybrid system enables improvement of photocatalytic biohydrogen production in the presence of oxygen. Int. J. Hydrog. Energy.

[34] Abdel A.M.S., Salama H.E. (2021). Developing multifunctional edible coatings based on alginate for active food packaging. Int. J. Biol. Macromol..

[35] Pratiksha S., Pankaj B., Omprakash S.Y. (2020). Synthesis, characterization and application of crosslinked alginate as green packaging material. Heliyon.

[36] Sikorski P., Mo F., Skjak B.G., Stokke B.T. (2007). Evidence for egg-box-compatible interactions in calcium-alginate gels from fiber X-ray diffraction. Biomacromolecules.

[37] Kumar A., Belhaj M., DiPette D.J., Potts J.D. (2021). A Novel Alginate-Based Delivery System for the Prevention and Treatment of Pressure-Overload Induced Heart Failure. Front. Pharmacol..

[38] Oki Y., Kirita K., Ohta S., Ohba S., Horiguchi I., Sakai Y., Ito T. (2019). Switching of Cell Proliferation/ Differentiation in Thiol-Maleimide Clickable Microcapsules Triggered by in Situ Conjugation of Biomimetic Peptides. Biomacromolecules.

[39] Ambaye T.G., Vaccari M., Prasad S., van Hullebusch E.D., Rtimi S. (2022). Preparation and applications of chitosan and cellulose composite materials. J. Environ. Manag..

[40] Zhao M.G., He H., Guo D.J., Zhang X., Jia L., Hou T., Ma A.M. (2022). Chitosan oligosaccharides-tripolyphosphate microcapsules as efficient vehicles for desalted duck egg white peptides-calcium: Fabrication, entrapment mechanism and in vivo calcium absorption studies. LWT-Food Sci. Technol..

[41] Li Z.L., Chen P., Xu X.Z., Ye X., Wang J. (2009). Preparation of chitosan-sodium alginate microcapsules containing ZnS nanoparticles and its effect on the drug release. Mater. Sci. Eng. C.

[42] Salvatore D.G., Chasper P., Lipps G. (2020). Stable and selective permeable hydrogel microcapsules for high-throughput cell cultivation and enzymatic analysis. Microb. Cell Factories.

[43] Ansari Z., Goomer S. (2022). Natural Gums and Carbohydrate-Based Polymers: Potential Encapsulants. Indo Glob. J. Pharm. Sci..

[44] Alicia H., Fabra M.J., Frédéric D., Cécile D.B., Andrée V. (2009). Interface and aroma barrier properties of iota-carrageenan emulsion–based films used for encapsulation of active food compounds. J. Food Eng..

[45] Joanna T., Ewelina J., Ewa P., Barbara B., Joanna K.D. (2019). Furcellaran-Coated Microcapsules as Carriers of Cyprinus carpio Skin-Derived Antioxidant Hydrolysate: An In Vitro and In Vivo Study. Nutrients.

[46] Raú l.E.C., Pablo R.S., Adriana N.M., Silvina R.D. (2020). Pyropia columbina phycocolloids as microencapsulating material improve bioaccessibility of brewers’ spent grain peptides with ACE-I inhibitory activity. Int. J. Food Sci. Technol..

[47] Nazia T., Suhani D.K. (2020). Synthesis, characterization and applications of copolymer of β-cyclodextrin: A review. J. Polym. Res..

[48] Crini G., Fourmentin S., Fenyvesi É., Torri G., Fourmentin M., Morin C.N. (2018). Cyclodextrins, from molecules to applications. Environ. Chem. Lett..

[49] Pawar S., Shende P. (2020). A Comprehensive Patent Review on β-cyclodextrin Cross-linked Nanosponges for Multiple Applications. Recent Pat. Nanotechnol..

[50] Desai D., Shende P. (2021). Monodispersed cyclodextrin-based nanocomplex of neuropeptide Y for targeting MCF-7 cells using a central composite design. J. Drug Deliv. Sci. Technol..

[51] Chen L.Y., Gabriel E.R., Muriel S. (2006). Food protein-based materials as nutraceutical delivery systems. Trends Food Sci. Technol..

[52] Dave J., Ye X., Jethro M., Xiao H. (2017). Protein-Based Drug-Delivery Materials. Materials.

[53] Fan Q.Q., Ma J.Z., Xu Q., Zhang J., Demetra S., Gaidău C., Guo C. (2015). Animal-derived natural products review: Focus on novel modifications and applications. Colloids Surf. B Biointerfaces.

[54] Kantrol K.S., Monika K., Ravi S.P. (2019). Chylomicron mimicking solid lipid nanoemulsions encapsulated enteric minicapsules targeted to colon for immunization against hepatitis B. Int. Immunopharmacol..

[55] Ahmady A., Hayati A.S.N. (2021). A review: Gelatine as a bioadhesive material for medical and pharmaceutical applications. Int. J. Pharm..

[56] Favaro C.S., Santana A.S., Monterrey E.S., Trindade M.A., Netto F.M. (2009). The use of spray drying technology to reduce bitter taste of casein hydrolysate. Food Hydrocoll..

[57] Niu H.X., Chang J., Jia Y.D. (2015). Microencapsulation of crystalline-methionine enclosed with gelatine and sodium alginate by spray-drying. Mater. Res. Innov..

[58] Ashaolu T.J. (2020). Applications of soy protein hydrolysates in the emerging functional foods: A review. Int. J. Food Sci. Technol..

[59] Gao X.Q., Xiong G.Y., Fu L., Liu S.L. (2019). Water distribution of raw and heat-induced gelation of minced pork paste prepared by soy protein isolates and carrageenan: Ingredients modify the gelation of minced pork. J. Food Process. Preserv..

[60] Wei C.L. (2019). Construction of soybean peptide-curcumin nanoparticles and their microencapsulation. South China Univ. Technol..

[61] Zhao C.H., Chen N., Ashaolu T.J. (2022). Whey proteins and peptides in health-promoting functions—A review. Int. Dairy J..

[62] Farizano J.V., Díaz V.L.I., Masias E., Baillo A.A., Torino M.I., Fadda S., Vanden B.N.L., Montenegro M.A., Saavedra L., Minahk C. (2022). Biotechnological use of dairy by-products for the production and microencapsulation of the food preservative enterocin CRL35. FEMS Microbiol. Lett..

[63] Zubair M., Pradhan R.A., Arshad M., Ullah A. (2021). Recent Advances in Lipid Derived Bio-Based Materials for Food Packaging Applications. Macromol. Mater. Eng..

[64] Blanco-Pascual N., Koldeweij R.B.J., Stevens R.S.A., Montero M.P., Gómez-Guillén M.C., Cate A.T. (2014). Peptide Microencapsulation by Core-Shell Printing Technology for Edible Film Application. Food Bioprocess Technol..

[65] Jiang Z.L., To N. (2022). Recent advances in chemically modified cellulose and its derivatives for food packaging applications: A review. Polymers.

[66] Aomatsu Y., Nakajima Y., Ohyama T., Kin T., Kanehiro H., Hisanaga M., Ko S., Nagao M., Tatekawa Y., Sho M. (2000). Efficacy of agarose/polystyrene sulfonic acid microencapsulation for islet xenotransplantation. Transplant. Proc..

[67] Nishimura M., Iizuka N., Fujita Y., Sawamoto O., Matsumoto S. (2017). Effects of encapsulated porcine islets on glucose and C-peptide concentrations in diabetic nude mice 6 months after intraperitoneal transplantation. Xenotransplantation.

[68] Cesar A.R.B., Larissa P.P., Elisabete A.L.G., Nilce M.S., Priscilla A.B.M.L., Douglas D.A.S., Andreia B.M., Marlus C., Eduardo F.V. (2021). HPMCP-coated microcapsules containing the ctx (Ile21)-ha antimicrobial peptide reduce the mortality rate caused by resistant salmonella enteritidis in laying hens. Antibiotics.

[69] Jenny K.R., Luz S., Mary A.A. (2006). Stabilization of oils by microencapsulation with heated protein-glucose syrup mixtures. J. Am. Oil Chem. Soc..

[70] Pavel S., Vladimir M. (2019). Protein interaction with charged macromolecules: From model polymers to unfolded proteins and post- translational modifications. Int. J. Mol. Sci..

[71] Swati K., Aasima R., Savita S. (2017). Protein engineering and its applications in food industry. Taylor Fr..

[72] Wang Z.G., Ju X.R., He R., Yuan J., Wang L.F. (2015). Effect of rapeseed protein structural modification on microstructural properties of peptide microcapsules. Food Bioprocess Technol..

[73] Deborah M.S., Joachim K. (2002). A synthetic polymer matrix for the delayed or pulsatile release of water-soluble peptides. J. Control. Release.

[74] Li X.M., Xu Y.L., Chen G.G., Wei P., Ping Q.N. (2008). PLGA nanoparticles for the oral delivery of 5-Fluorouracil using high pressure homogenization-emulsification as the preparation method and in vitro/in vivo studies. Drug Dev. Ind. Pharm..

[75] Justin K.Y.H., Steven P.S. (2020). Characterization of octreotide-PLGA binding by isothermal titration calorimetry. Biomacromolecules.

[76] Lim S.M., Eom H.N., Jiang H.H., Sohn M.J., Lee K.C. (2015). Evaluation of PEGylated exendin-4 released from poly (lactic-co-glycolic acid) microspheres for antidiabetic therapy. J. Pharm. Sci..

[77] Zhang Y., Wu X.H., Han Y.R., Mo F., Duan Y.R., Li S.M. (2010). Novel thymopentin release systems prepared from bioresorbable PLA-PEG-PLA hydrogels. Int. J. Pharm..

[78] Burdock G.A., Flamm W.G. (1999). A review of the studies of the safety of polydextrose in food. Food Chem. Toxicol..

[79] Marília P.F., Bruna G., Maria E.C.S., Izabela D.A., Maria T.B.P. (2019). Microencapsulation performance of Fe-peptide complexes and stability monitoring. Food Res. Int..

[80] Günay K.A., Berthier D.L., Jerri H.A., Benczédi D., Klok H.-A., Herrmann A. (2017). Selective Peptide-Mediated Enhanced Deposition of Polymer Fragrance Delivery Systems on Human Hair. ACS Appl. Mater. Interfaces.

[81] Shashi K.B., Ranjit G., Choi T.R., Jung H.R., Yang S.Y., Song H.S., Jeon J.M., Kim J.S., Lee Y.K., Yang Y.H. (2019). Poly(3-hydroxybutyrate-co-3-hydroxyhexanoate) production from engineered Ralstonia eutropha using synthetic and anaerobically digested food waste derived volatile fatty acids. Int. J. Biol. Macromol..

[82] Sharma V., Sehgal R., Gupta R. (2020). Polyhydroxyalkanoate (PHA): Properties and Modifications. Polymer.

[83] Cao L.D., Liu Y.J., Xu C.L., Zhou Z.L., Zhao P.Y., Niu S.J., Huang Q.L. (2019). Biodegradable poly(3-hydroxybutyrate-co-4-hydroxybutyrate) microcapsules for controlled release of trifluralin with improved photostability and herbicidal activity. Mater. Sci. Eng. C.

[84] Svetlana U., Cruz M.J., Cabeza L.F., Grágeda M. (2016). Preparation and Characterization of Inorganic PCM Microcapsules by Fluidized Bed Method. Materials.

[85] Jin Y., Zhou Q., Li Z.H., Yang Z.H., Fan H.J. (2020). Calcium-cross linked polysaccharide microcapsules for controlled release and antimicrobial applications. Colloids Surf. A Physicochem. Eng. Asp..

[86] Chatterjee S., Judeh Z.M.A. (2016). Impact of encapsulation on the physicochemical properties and gastrointestinal stability of fish oil. LWT-Food Sci. Technol..

[87] Sahar A., Elham A., Iman K., Seid M.J. (2018). Lipid nano scale cargos for the protection and delivery of food bioactive ingredients and nutraceuticals. Trends Food Sci. Technol..

[88] Beghetto V., Sole R., Buranello C., AlAbkal M., Facchin M. (2021). Recent advancements in plastic packaging recycling: A mini-review. Materials.

[89] Gou M.L., Wei X.W., Men K., Wang B.L., Luo F., Zhao X., Wei Y.Q., Qian Z.Y. (2011). PCL/PEG copolymeric nanoparticles: Potential nanoplatforms for anticancer agent delivery. Curr. Drug Targets.

[90] Kim M.R., Feng T., Zhang Q., Chan H.Y.E., Chau Y. (2019). Co-Encapsulation and Co-Delivery of Peptide Drugs via Polymeric Nanoparticles. Polymers.

[91] Zhang D.M., Zhang Q., Lu Y.L., Yao Y., Li S., Jiang J., Liu G.L., Liu Q.J. (2016). Peptide Functionalized Nanoplasmonic Sensor for Explosive Detection. Nano-Micro Lett..

[92] Song J.Y., CortezJugo C., Shirbin S.J., Lin Z.X., Pan S.J., Qiao G.G., Caruso F. (2022). Immobilization and Intracellular Delivery of Structurally Nanoengineered Antimicrobial Peptide Polymers Using Polyphenol-Based Capsules. Adv. Funct. Mater..

[93] Miléna L., Nikolett K.S., Vince A., András J.L., István A. (2019). Microparticles, Microspheres, and Microcapsules for Advanced Drug Delivery. Sci. Pharm..

[94] Ghiman R., Pop R., Rugina D., Focsan M. (2022). Recent progress in preparation of microcapsules with tailored structures for bio-medical applications. J. Mol. Struct..

[95] Jaganathan M., Madhumitha D., Dhathathreyan A. (2014). Protein microcapsules: Preparation and applications. Adv. Colloid Interface Sci..

[96] Alexander G., Martin W., Andreas B.S. (2010). Oral peptide delivery: In-vitro evaluation of thiolated alginate/poly(acrylic acid) microparticles. J. Pharm. Pharmacol..

[97] Tang Y.T., Arbaugh B., Park H., Scher H.B., Bai L., Mao L., Jeoh T. (2022). Targeting enteric release of therapeutic peptides by encapsulation in complex coacervated matrix microparticles by spray drying. J. Drug Deliv. Sci. Technol..

[98] Raúl E.C., Andrea C.S., Luis C.G., Silvina R.D., David B.A. (2019). Bioactive Phaseolus lunatus peptides release from maltodextrin/gum arabic microcapsules obtained by spray drying after simulated gastrointestinal digestion. Int. J. Food Sci. Technol..

[99] Situ W.B., Li X.X., Liu J., Chen L. (2015). Preparation and characterization of glycoprotein-resistant starch complex as a coating material for oral bioadhesive microparticles for colon-targeted polypeptide delivery. J. Agric. Food Chem..

[100] Situ W.B., Chen L., Wang X.Y., Li X.X. (2014). Resistant starch film-coated microparticles for an oral colon-specific polypeptide delivery system and its release behaviors. J. Agric. Food Chem..

[101] Agrawal H., Joshi R., Gupta M. (2021). Optimization of pearl millet-derived bioactive peptide microspheres with double emulsion solvent evaporation technique and its release characterization. Food Struct..

[102] Wu Z.M., Zhou L.Y., Guo X.D., Jiang W., Ling L., Qian Y., Luo K.Q., Zhang L.J. (2012). HP55-coated capsule containing PLGA/RS nanoparticles for oral delivery of insulin. Int. J. Pharm..

[103] Kondiah P.P.D., Choonara Y.E., Tomar L.K., Tyagi C., Kumar P., Toit L.C., Marimuthu T., Modi G., Pillay V. (2017). Development of a Gastric Absorptive, Immediate Responsive, Oral Protein-Loaded Versatile Polymeric Delivery System. AAPS PharmSciTech.

[104] Sun X.T., Liu M., Xu Z.R. (2014). Microfluidic fabrication of multifunctional particles and their analytical applications. Talanta.

[105] Shimanovich U., Levin A., Eliaz D., Michaels T., Toprakcioglu Z., Frohm B., De G.E., Linse S., ÅkerfeldT K.S., Knowles T.P.J. (2021). pH-Responsive capsules with a fibril scaffold shell assembled from an amyloidogenic peptide. Small.

[106] Han F.Y., Xu W.Z., Kumar V., Cui C.S., Li X.R., Jiang X.Y., Woodruff T.M., Whittaker A.K., Smith M.T. (2021). Optimisation of a Microfluidic Method for the Delivery of a Small Peptide. Pharmaceutics.

[107] Calva E.S.J., Lugo C.E., Jiménez F.M. (2019). Microencapsulation of cocoa liquor nanoemulsion with whey protein using spray drying to protection of volatile compounds and antioxidant capacity. J. Microencapsul..

[108] Zhang C., Siew L.A.K., Chen X.D., Siew Y.Q. (2020). Microencapsulation of fermented noni juice via micro-fluidic-jet spray drying: Evaluation of powder properties and functionalities. Powder Technol..

[109] Miriam G.V., Hugo E.A., Guadalupe M.G., Hugo E.S., Enrique A.G. (2019). Oxidative stability of green coffee oil (Coffea arabica) microencapsulated by spray drying. Processes.

[110] Alfonso G.G., Elena C.H., María F.M., Lucía M.B. (2015). Massive, generic, and controlled microencapsulation by flow focusing: Some physicochemical aspects and new applications. J. Flow Chem..

[111] Laura A., Graça F., Claúdia M., Joana V., Isabel C. (2014). Gouveia. Bioactive microsphere-based coating for biomedical-textiles with encapsulated antimicrobial peptides (AMPs). Ciência Tecnol. Dos Mater..

[112] Jaime M.G., Teresa G.M., Juan C.M., Juan M. (2018). Yeast Immobilization Systems for Alcoholic Wine Fermentations: Actual Trends and Future Perspectives. Front. Microbiol..

[113] Huizar C.C., Ji N., Reddick R., Ostroff G.R., Forsthuber T.G. (2021). Glucan particles as a novel adjuvant for the induction of experimental autoimmune encephalomyelitis. Cell. Immunol..

[114] Mochizuki Y., Kogawa R., Takegami R., Nakamura K., Wakabayashi A., Ito T., Yoshioka Y. (2020). Co-microencapsulation of islets and msc cellsaics, mosaic-like aggregates of mscs and recombinant peptide pieces, and therapeutic effects of their subcutaneous transplantation on diabetes. Biomedicines.

[115] Jiang T., Singh B.J., Sushila M., Li H.S., Kang S.K., Bok J.D., Cho C.G., Choi Y.J. (2014). Oral delivery of probiotic expressing M cell homing peptide conjugated BmpB vaccine encapsulated into alginate/chitosan/alginate microcapsules. Eur. J. Pharm. Biopharm..

[116] Wang Y., Hou L.L., Su D., Wang Z.X., Jia Y.M. (2021). Optimization of Brevilaterin Microencapsulation and Analysis of Slow-release Characteristics. Food Sci..

[117] Hu Y.Q., Chen Y., Lin L.J., Zhang J.H., Lan R.G., Wu B.L. (2021). Studies on antimicrobial peptide-loaded nanomaterial for root caries restorations to inhibit periodontitis related pathogens in periodontitis care. J. Microencapsul..

[118] Cesar R.A., Saraiva M.M., Daniel F.M. (2022). Alginate-based microparticles coated with HPMCP/AS cellulose-derivatives enable the Ctx(Ile 21)-Ha antimicrobial peptide application as a feed additive. Int. J. Biol. Macromol..

